# Effect of a retinoid X receptor partial agonist on airway inflammation and hyperresponsiveness in a murine model of asthma

**DOI:** 10.1186/s12931-017-0507-z

**Published:** 2017-01-23

**Authors:** Utako Fujii, Nobuaki Miyahara, Akihiko Taniguchi, Naohiro Oda, Daisuke Morichika, Etsuko Murakami, Hikari Nakayama, Koichi Waseda, Mikio Kataoka, Hiroki Kakuta, Mitsune Tanimoto, Arihiko Kanehiro

**Affiliations:** 1 Department of Hematology, Oncology, Allergy and Respiratory Medicine, Okayama University Graduate School of Medicine, Dentistry, and Pharmaceutical Sciences, 2-5-1 Shikata-cho, Kita-ku, Okayama, 700-8558 Japan; 2 Division of Pharmaceutical Sciences, Okayama University Graduate School of Medicine, Dentistry, and Pharmaceutical Sciences, 1-1-1 Tsushima-naka, Kita-ku, Okayama, 700-8530 Japan

**Keywords:** Asthma, RXR, RXR partial agonist, NF-κB, Nitric oxide, Animal model

## Abstract

**Background:**

Retinoid X receptors (RXRs) are members of the nuclear receptor (NR) superfamily that mediate signaling by 9-cis retinoic acid, a vitamin A (retinol) derivative. RXRs play key roles not only as homodimers but also as heterodimeric partners—e.g., retinoic acid receptors (RARs), vitamin D receptors (VDRs), liver X receptors (LXRs), and peroxisome proliferator-activated receptors (PPARs). The NR family was recently associated with allergic diseases, but the role of RXRs in allergen-induced airway responses is not well defined.

The goal of this study is to elucidate the role of RXRs in asthma pathogenesis and the potency of RXR partial agonist in the treatment of allergic airway inflammation and airway hyperresponsiveness using a murine model of asthma.

**Methods:**

We investigated the effect of a novel RXR partial agonist (NEt-4IB) on the development of allergic airway inflammation and airway hyperresponsiveness (AHR) in a murine model of asthma. Balb/c mice were sensitized (days 0 and 14) and challenged (days 28–30) with ovalbumin (OVA), and airway inflammation and airway responses were monitored 48 h after the last OVA challenge. NEt-4IB was administered orally on days 25 to 32.

**Results:**

Oral administration of NEt-4IB significantly suppressed AHR and inflammatory cell accumulation in the airways and attenuated the levels of TNF-α in the lung and IL-5, IL-13 and NO levels in bronchoalveolar lavage (BAL) fluid and the number of periodic acid Schiff (PAS)-positive goblet cells in lung tissue. Treatment with NEt-4IB also significantly suppressed NF-κB expression.

**Conclusion:**

These data suggest that RXRs may be of crucial importance in the mechanism of allergic asthma and that the novel RXR partial agonist NEt-4IB may be a promising candidate for the treatment of allergic airway inflammation and airway hyperresponsiveness in a model of allergic asthma.

## Background

Asthma is characterized by chronic airway inflammation and airway hyperresponsiveness (AHR). Airway inflammation results from the influx of activated eosinophils and T cells at the site of inflammation. T cells, particularly T helper (Th) type 2 cells, which release IL-4, IL-5, and IL-13, play a pivotal role in the development of AHR and eosinophilic inflammation [1, 2]. However, asthma is a heterogeneous disease with a complex pathophysiology. Current management based on inhaled corticosteroids and long-acting β2 agonists is effective in controlling asthma in most patients, but some patients develop severe asthma.

Retinoid X receptors (RXRs) are nuclear receptors that control gene transcription dependent on ligand binding [3] and act as homodimers or heterodimers with peroxisome proliferator-activated receptors (PPARs), liver X receptors (LXRs), and other nuclear receptors (NRs) [4–6]. Therefore, RXR agonists have multipotent effects on the mechanism of airway inflammation and may play a critical and powerful role in the treatment of heterogeneous asthma. However, the relative proportions of RXRs interacting as homodimers or heterodimers remain unclear because RXRs are thought to function “permissively” as immune modulators.

RXR agonists are effective in the treatment of several diseases [7]. For example, the RXR full agonist Bexarotene is used for the treatment of cutaneous T cell lymphoma [8] and is also effective against type 2 diabetes, metabolic syndrome [9], Alzheimer’s disease [10], and Parkinson’s disease in animal models [11]. Kakuta et al. reported that the novel RXR full agonist NEt-TMN is potentially effective to reduce adverse effects while retaining desired activities [12]. However, RXR full agonists, including NEt-TMN and Bexarotene, are associated with strong adverse events, such as weight gain, hepatomegaly, and blood triglyceride elevation, in the treatment of underlying diseases [12, 13].

To avoid these severe adverse events, we considered that moderate activation of RXR might be sufficient to regulate allergic inflammation, and we focused on the production of new RXR partial agonists with lower maximum activation of RXR compared to RXR full agonists. The novel RXR partial agonist NEt-4IB has a maximum 55% RXR efficacy compared with full agonists in a luciferase reporter gene assay, and 28 consecutive days’ administration of NEt-4IB resulted in no significant adverse events [14]. Furthermore, we have investigated the pharmacokinetics of NEt-4IB in vivo by positron emission tomography (PET) and revealed an increase in radioactivity in PET imaging of the lung [15].

Other NR superfamily members have been related to allergic inflammation. A PPARγ agonist reduces the response to cockroach allergen challenge in a murine asthma model [16]. An LXR agonist reduces the response to OVA-allergen challenge in a murine model of asthma [17]. NR4A attenuates airway inflammation in a mouse model of asthma [18]. These data suggest that RXRs, which act as both homodimers and heterodimers with other NRs, may be potential targets in the prevention and management of heterogeneous asthma patients. Therefore, we investigated for the first time the effect of the RXR partial agonist NEt-4IB on the development of AHR and eosinophilic airway inflammation in OVA-sensitized and -challenged mice.

## Methods

### Animals

Female BALB/c mice at 8–12 weeks of age were purchased from Charles River Japan, Inc. (Yokohama, Japan). The mice were maintained on an OVA-free diet. All experimental animals used in the present study were housed under a protocol approved by the Institutional Animal Care and Use Committee of Okayama University Medical School (Okayama, Japan).

### Sensitization and airway challenge

The mice were sensitized by intraperitoneal injection of 20 μg of OVA (Grade V; Sigma-Aldrich, St Louis, MO, USA) emulsified in 2.25 mg of alum (Inject Alum; Pierce Biotechnology, Rockford, IL, USA) on days 0 and 14. The mice were subsequently challenged with OVA (1% in saline) by ultrasonic nebulization for 20 min on days 28, 29, and 30 (OVA/OVA). AHR was assessed 48 h after the last challenge, and tissues and cells were obtained for further analysis [19]. Control mice were non-sensitized but challenged with OVA (PBS/OVA).

### Administration of the novel RXR partial agonist and prednisone

The mice received the novel RXR partial agonist (NEt-4IB) (0.0015, 0.005, and 0.015%) or prednisone (PSL) (0.005%) daily on days 25 to 32 orally. The control mice received vehicle.

### Determination of airway responsiveness

A flexiVent small-animal ventilator (SCIREQ, Montreal, PQ, Canada) was used to assess airway function (Snapshot) in anesthetized (intraperitoneal injection of sodium pentobarbital, 70 mg/kg), mechanically ventilated animals. Changes in lung resistance (RL) were measured in response to increasing doses of inhaled methacholine (MCh) [20]. Airway responsiveness was assessed as the change in airway function (150 breaths/min, tidal volume: 10 ml/kg) after challenge with aerosolized MCh administered for 10 s (60 breaths/min, tidal volume: 20 ml/kg) at increasing concentrations (0, 1.56, 6.25, 12.5 and 25 mg/ml) [21]. Baseline RL values in response to saline at 48 h were first determined. RL data were continuously collected for up to 3 min, and the maximum values were obtained.

### BAL fluid

After the assessment of AHR, the lungs were lavaged with Hanks’ balanced salt solution via the tracheal tube (1 ml; 37 °C). The number of cells in the bronchoalveolar lavage (BAL) fluid was determined. Cytospin slides were stained and differentiated in a blinded manner by counting at least 200 cells under light microscopy.

### Histological studies of the lung

Lungs were fixed in 10% formalin. Blocks of lung tissue were cut around the main bronchus and embedded in paraffin blocks. Lung sections with a thickness of 4 μm were stained with hematoxylin-eosin (H&E) to analyze the difference between eosinophils and neutrophils, and periodic acid Schiff (PAS) was used to identify mucus-containing cells (goblet cells) as previously described [22].

### Measurement of cytokines, chemokines and nitric oxide (NO)

The cytokine levels in the BAL fluid and homogenized lung were measured by ELISA as previously described [23]. All cytokine and chemokine ELISAs (R&D Systems, Minneapolis, MN, USA) were performed according to the manufacturer’s directions. The limits of detection were 5 pg/ml for IL-4, 0.68 pg/ml for IL-5, 2 pg/ml for IL-13, and 0.36 pg/ml for tumor necrosis factor alpha (TNF-α). For the preparation of lung homogenates, lung tissue was frozen at −70 °C immediately after euthanasia. Next, the lung tissue was mixed with a PBS-0.1% Triton-X100 solution containing proteinase inhibitors at a 1:2.5 ratio of weight per volume (Sigma-Aldrich). The specimens were homogenized and then centrifuged at 14,000 rpm for 30 min. The supernatants were frozen at −70 °C until analysis. NO with BAL fluid was measured using a nitrate/nitrite fluorometric assay kit (Cayman chemical, Ann Arbor, MI, USA) as previously described [24].

### Cell preparation and culture

Lung mononuclear cells (MNCs) from OVA-sensitized and -challenged mice were isolated as previously described following collagenase digestion [22]. Lung MNCs were cultured for 24 h in 96-well round-bottom plates in the presence or absence of OVA (10 μg/ml). Ten days after OVA-sensitization, the spleens were removed from mice and placed in PBS. The tissue was dispersed into single-cell suspensions, and spleen MNCs were purified by Ficoll-Hypaque gradient centrifugation (Sigma-Aldrich). The cells (4 × 10^5^) were then cultured for 48 h in 96-well round-bottom plates in the presence or absence of OVA (10 μg/ml) and drugs (NEt-4IB or PSL) as previously described [25]. The cytokine levels in the culture supernatants were measured by ELISA.

### Measurements of blood parameters

Triglyceride (TG) levels were measured using a Fuji Dry Chem system (Dry Chem 4000 V; Fuji Medical Co., Tokyo, Japan).

### Flow cytometry

After lung cell purification, intracytoplasmic cytokine staining was performed as previously described [26, 27]. The cells were stained for cell surface markers with PE-conjugated anti-CD4 (BD Biosciences, Franklin Lakes, NJ, USA), FITC-conjugated anti-CD8 (BD Biosciences), or APC-conjugated anti-CD3 (BD Biosciences) and then analyzed using a MACS Quant flow cytometer (Miltenyi Biotec, Auburn, CA, USA) with FlowJo software (TreeStar, Ashland, OR, USA). The numbers of cytokine-producing CD4^+^ or CD8^+^ T cells per lung were calculated from the percentages of cytokine-producing cells and the numbers of CD4^+^ or CD8^+^ T cells isolated from the lung. The cells were also stained for cell surface markers with APC-conjugated anti-CD11b (BD Biosciences) and FITC-conjugated anti-CD11c (BD Biosciences).

### Total RNA isolation and quantitative real-time PCR

The left lung was homogenized, and total RNA was extracted using TRIzol reagent (Invitrogen, Carlsbad, CA, USA), followed by treatment with DNase (Qiagen, Valencia, CA, USA) according to the manufacturer’s instructions. Reverse transcription was performed using oligo (dT) primers and Invitrogen Superscript II Reverse Transcriptase (Life Technologies, Grand Island, NY, USA) to obtain cDNA for PCR. Quantitative real-time PCR was performed in a 25-μl reaction using FastStart Essential DNA Green Master (Roche, Basel, Switzerland). The primers sequences were as follows: NF-κB, forward, 5-CAACAGATGGGCTACACAGAGG-3, and reverse, 5-GGAAGACGAGAGAGGCAGACA-3; GAPDH, forward, 5-TATGTCGTGGAGTCTACTGGT-3, and reverse, 5-GAGTTGTCATATTTCTCGTGG-3. The relative expression levels of each target were normalized to GAPDH and calculated using the ΔΔ cycle threshold method. There were no differences in GAPDH expression among the groups [28].

### Immunoblotting

Protein extracts were prepared from homogenates suspended in PBS-0.1% Triton-X100 solution containing proteinase inhibitors (BD Biosciences) and centrifuged at 14,000 rpm min at 4 °C. After quantification using the Bio-Rad protein assay (Bio-Rad, Hercules, CA, USA), SDS was added to the supernatant. Aliquots of the supernatant, each containing 30 μg of protein, were separated by SDS-PAGE and electrically transferred to nitrocellulose membranes. Nonspecific binding sites were blocked with Tris-buffered saline (TBS) containing 5% nonfat dry powdered milk (wt/vol) for 1 h at room temperature. After a brief rinse with TBS containing 0.1% Tween 20 (TBST), the protein blots were incubated with 1:50,000 diluted anti-NF-κB antibody (cat. no. ab32536, Abcam, Cambridge, MA, USA), 1:1000 diluted anti-inducible nitric oxide synthase antibody (cat. no. ab178945, Abcam) and 1:5000 diluted anti-β-actin (cat. no. ab8227, Abcam) overnight at 4 °C. The secondary antibody was anti-rabbit IgG (horseradish peroxidase-linked, species-specific whole antibodies; GE Healthcare, Buckinghamshire, UK), which was used at a 1:5000 dilution.

### Statistical analysis

All results are expressed as the mean ± standard error of the mean (SEM). ANOVA was used to determine the levels of difference among all groups. Pairs of groups of samples distributed parametrically were compared by unpaired two-tailed Student’s *t*-test, and those samples distributed nonparametrically were compared by the Mann–Whitney *U* test. Significance was assumed at *P* values <0.05.

## Results

### Treatment with a novel RXR partial agonist attenuates AHR and airway inflammation in OVA-sensitized and -challenged mice

**Fig. 1:**
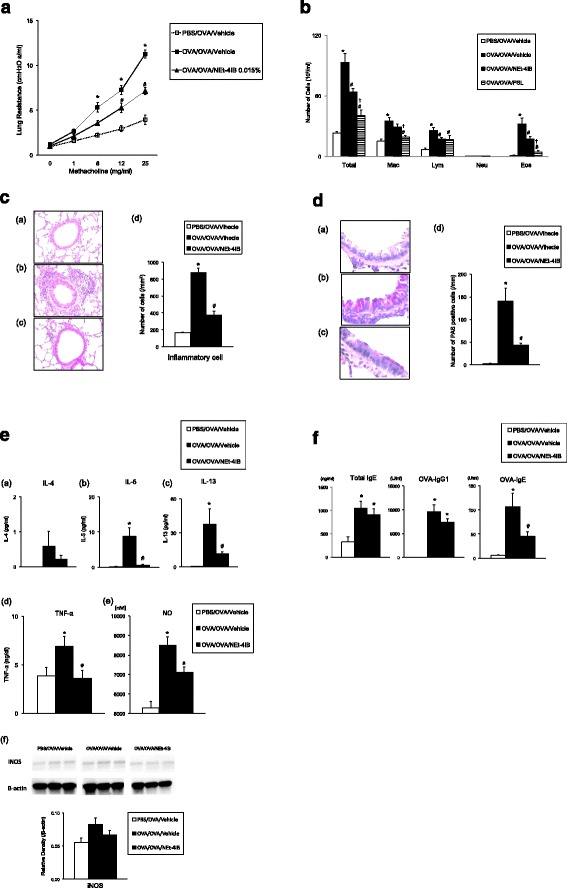
Treatment with NEt-4IB and PSL attenuates AHR and airway inflammation. **a** AHR. **b** Cell composition in BAL fluid. **c** H&E-stained lung tissue (final magnification: ×400). (*a*) PBS/OVA/Vehicle, (*b*) OVA/OVA/Vehicle, (*c*) OVA/OVA/NEt-4IB, (*d*) inflammatory cell numbers in the peribronchial and perivascular tissue. **d** Treatment with NEt-4IB suppresses goblet cell metaplasia (final magnification: ×1000). (*a*) PBS/OVA/Vehicle, (*b*) OVA/OVA/Vehicle, (*c*) OVA/OVA/NEt-4IB, (*d*) the number of mucus-positive cells. **e** (*a*) IL-4, (*b*) IL-5, (*c*) IL-13 in BAL fluid, (*d*) TNF-α in the lung, (*e*) NO in BAL fluid. *n* = 8–20 in each group. (*f*) The expression levels of iNOS in the lung were determined by Western blotting as described in the Methods. *n* = 4 in each group. **f** Serum levels of (*a*) total IgE, (*b*) anti-OVA IgG1, (*c*) anti-OVA IgE. *n* = 8–20 in each group. The results for each group are expressed as the mean ± SEM. *Mac* macrophage, *Eos* eosinophil, *Lym* lymphocyte, and *Neu* neutrophil. * Significant differences (*P* < 0.05) between PBS/OVA/Vehicle and OVA/OVA/Vehicle. # Significant differences (*P* < 0.05) between OVA/OVA/Vehicle and OVA/OVA/NEt-4IB. † Significant difference (*P* < 0.05) between OVA/OVA/NEt-4IB and OVA/OVA/PSL

We assessed the AHR of OVA-sensitized and -challenged mice treated with vehicle (OVA/OVA/vehicle) to increasing doses of inhaled MCh. OVA/OVA/vehicle mice developed AHR compared with the non-sensitized but OVA-challenged mice treated with vehicle (PBS/OVA/vehicle). The administration of the novel RXR partial agonist (NEt-4IB) significantly attenuated the increase in AHR throughout the MCh dose–response curve in a dose-dependent manner (Table 1, Fig. 1A) and prevented the increase in the number of lymphocytes and eosinophils in the BAL fluid compared with OVA/OVA/vehicle mice (Fig. 1B).

**Table 1:** Dose-dependent effects of NEt-4IB and PSL on AHR

Methacholine (mg/mL)	0	1	6	12	25
PBS/OVA/Vehicle	0.93 ± 0.19	1.57 ± 0.15	2.22 ± 0.20	2.93 ± 0.3.3	3.95 ± 0.53
OVA/OVA/Vehicle	1.19 ± 0.26	2.63 ± 0.51	5.31 ± 0.52*	7.25 ± 0.45*	11.27 ± 0.87*
OVA/OVA/NEt-4IB 0.0015%	0.82 ± 0.05	2.14 ± 0.31	4.26 ± 0.59	5.81 ± 0.73	9.93 ± 1.66
OVA/OVA/NEt-4IB 0.005%	0.84 ± 0.10	1.66 ± 0.20	3.97 ± 0.44	6.07 ± 0.41	8.75 ± 0.56
OVA/OVA/NEt-4IB 0.015%	1.00 ± 0.15	2.10 ± 0.25	3.57 ± 0.52	5.28 ± 0.37**	7.14 ± 0.31**
OVA/OVA/PSL 0.005%	0.89 ± 0.06	1.52 ± 0.10	3.13 ± 0.34**	4.73 ± 0.47**	6.38 ± 0.42**

The results for each group are expressed as the mean RL ± SEM (cmH_2_O s/ml) (*n* = 8–12 in each group). *Significant difference (*P* < 0.05) between PBS/OVA/Vehicle and OVA/OVA/Vehicle. **Significant difference (*P* < 0.05) between OVA/OVA/Vehicle and OVA/OVA/NEt-4IB or OVA/OVA/PSL

### Effects of prednisone on airway inflammation and hyperresponsiveness in OVA-sensitized and -challenged mice

PSL-treated mice exhibited a reduced number of macrophages and eosinophils in the BAL fluid compared to OVA/OVA/vehicle mice and OVA/OVA/NEt-4IB mice (Fig. 1B). The administration of PSL significantly attenuated the increase in AHR; however, interestingly, no significant difference was observed between OVA/OVA/PSL mice and OVA/OVA/NEt-4IB mice (Table 1).

### Localization of inflammatory cells and mucus production in lung tissue

The development of AHR is associated with inflammatory changes in lung tissue [23]. To determine if treatment with NEt-4IB affected inflammatory changes in the lung, we assessed tissue inflammation. Hematoxylin-eosin-stained lung tissue revealed significant increases in inflammatory cells in peribronchial inflammation in OVA/OVA/vehicle mice compared with PBS/OVA/vehicle mice. Mice treated with NEt-4IB exhibited reduced numbers of eosinophils and lymphocytes in lung tissue (Fig. 1C). Lung sections were stained with PAS to identify mucus-containing cells in the airway epithelium. A significant increase in PAS-positive goblet cell hyperplasia was observed in OVA/OVA/vehicle mice. Treatment with NEt-4IB significantly reduced the number of PAS-positive cells per millimeter of basement membrane (Fig. 1D).

### Treatment with NEt-4IB suppresses IL-5, IL-13 and NO levels in BAL fluid and TNF-α and iNOS in the lung

Cytokine levels in BAL fluid and the lung were measured by ELISA. Treatment with vehicle resulted in significant increases in the levels of Th2 cytokines in BAL fluid and TNF-α in the lung. By contrast, treatment with NEt-4IB significantly reduced not only IL-5 and IL-13 levels but also TNF-α levels compared with vehicle-treated OVA/OVA mice (Fig. 1E a-d). IFN-γ was not detected even in OVA/OVA/vehicle mice in this study (data not shown).

In asthmatic patients, the level of nitric oxide, which is produced endogenously in the lung by nitric oxide synthase to elicit multiple physiological functions, can be measured in exhaled breath [29]. However, a deficiency in inducible nitric oxide synthase (iNOS) suppresses ozone-induced airway tissue injury and LPS-induced acute airway inflammation in mice [30, 31]. NO inhibitors block eosinophil recruitment in the lungs [32]. NO is also a potent vasodilator in the bronchial circulation and may play a major role in airway circulation [33]. In the present study, the level of NO in the BAL fluid was significantly suppressed in OVA/OVA NEt-4IB-treated mice compared with OVA/OVA vehicle-treated mice (Fig. 1E e), although a significant difference was not recognized among three groups in the expression levels of iNOS in the lung (Fig. 1E f).

### Treatment with the RXR partial agonist suppresses serum anti-OVA IgE antibody levels

The serum Ig levels in OVA/OVA/vehicle mice were elevated compared with those in PBS/OVA/vehicle mice, and treatment with NEt-4IB significantly suppressed OVA-specific IgE compared with OVA/OVA/vehicle mice (Fig. 1F).

### The numbers of CD4^+^ T cells, CD8^+^ T cells, CD11b^+^ cells, and CD11c^+^ cells in the lung

**Fig. 2:**
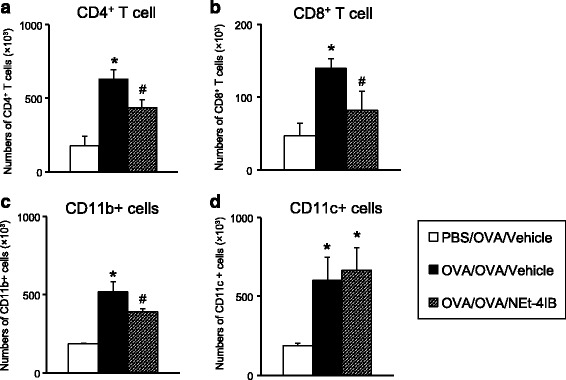
Numbers of CD4^+^, CD8^+^ T cells and CD11b^+^, CD11c^+^cells in the lung. The numbers of **a** CD4^+^, **b** CD8^+^ T cells, **c** CD11b^+^ cells, and **d** CD11c^+^ cells in the lung were calculated as described in the Methods section. *n* = 8–12 in each group. The results for each group are expressed as the mean ± SEM. * Significant differences (*P* < 0.05) between PBS/OVA/Vehicle and OVA/OVA/Vehicle. # Significant differences (*P* < 0.05) between OVA/OVA/Vehicle and OVA/OVA/NEt-4IB

To determine if the accumulation of T cells in the airways of sensitized and challenged mice was affected by treatment with NEt-4IB, lung cells were isolated, and the numbers of both CD4^+^ and CD8^+^ T cells were determined by flow cytometry. The numbers of CD4^+^ and CD8^+^ T cells were significantly lower in the lungs of NEt-4IB-treated mice compared to vehicle-treated mice (Fig. 2a, b). These data indicate that treatment with NEt-4IB attenuated the accumulation of CD4^+^ and CD8^+^ T cells in the airways of OVA-sensitized mice after challenge. The numbers of CD11b^+^ cells but not CD11c^+^ cells were significantly lower in the lungs of NEt-4IB-treated mice compared to vehicle-treated mice (Fig. 2c, d).

### Cytokine production by lung and spleen MNCs in vitro

**Fig. 3:**
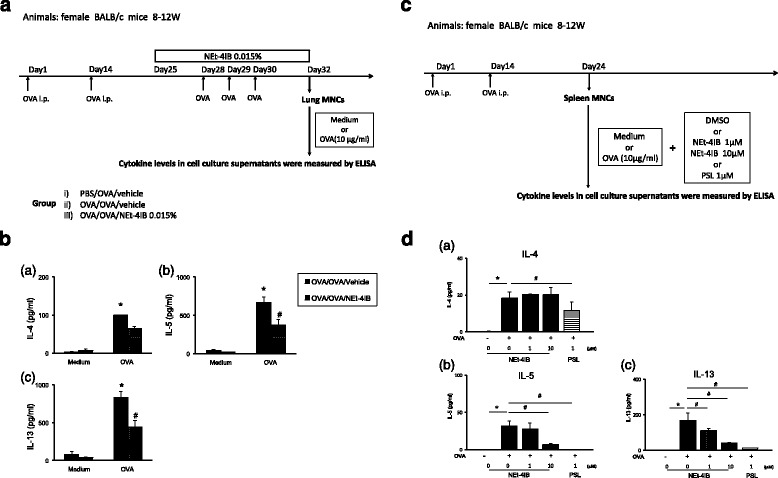
IL-4, IL-5, and IL-13 levels in culture supernatants from lung and spleen MNCs. **a** Experimental protocol. Lung mononuclear cells (MNCs) were isolated from OVA-sensitized and -challenged mice and were cultured for 24 h in the presence or absence of OVA (10 μg/ml) as previously described in the Methods [22]. **b** (*a*) IL-4, (*b*) IL-5, and (*c*) IL-13 levels in supernatants from cultured lung MNCs isolated from OVA-sensitized and -challenged mice in the presence or absence of OVA (10 μg/ml) were determined by ELISA. **c** Experimental protocol. Spleen MNCs were isolated from OVA-sensitized mice and were stimulated with OVA for 48 h in the presence or absence of NEt-4IB (1, 10 μM) or prednisone (1 μM) as previously described in the Methods [25]. **d** (*a*) IL-4, (*b*) IL-5, and (*c*) IL-13 levels in supernatants from cultured spleen MNCs isolated from OVA-sensitized mice in the presence or absence of OVA (10 μg/ml) and NEt-4IB (1, 10 μM) or PSL (1 μM) were determined by ELISA. *n* = 8–12 in each group. The results for each group are expressed as the mean ± SEM. * Significant differences (*P* < 0.05) between medium-stimulated OVA/OVA/Vehicle and OVA-stimulated OVA/OVA/Vehicle. # Significant differences between OVA-stimulated OVA/OVA/Vehicle and OVA/OVA/NEt-4IB or OVA/OVA/PSL

We assessed cytokine production from lung MNCs (Fig. 3A, B) and spleen MNCs (Fig. 3C, D) in vitro. Lung MNCs were isolated from OVA-sensitized and -challenged and vehicle mice (OVA/OVA/vehicle). Re-stimulation with OVA (10 μg/ml) for 24 h significantly increased Th2 cytokine levels in the lung MNC culture supernatants. However, in NEt-4IB-treated mice (OVA/OVA/NEt-4IB), both IL-5 and IL-13 levels were significantly suppressed in OVA-re-stimulated culture supernatants (Fig. 3B a-c). Next, spleen MNCs isolated 10 days after OVA sensitization in mice were stimulated with OVA (10 μg/ml) for 48 h in the presence or absence of NEt-4IB or PSL in vitro. The levels of Th2 cytokines were significantly increased in the culture supernatants from spleen MNCs after culture with OVA. Treatment with NEt-4IB (10 μM) significantly decreased the levels of IL-5 and IL-13 in the culture supernatants compared with the non-treated group in vitro. PSL (1 μM) had a significant effect on cytokine levels (Fig. 3D a-c).

### Triglyceride value in the blood

To assess adverse events associated with NEt-4IB, we measured serum TG levels, which were 331.3 ± 13.9, 332.1 ± 30.9, and 367.7 ± 48.5 ml/dL in PBS/OVA/vehicle, OVA/OVA/vehicle and OVA/OVA/NEt-4IB mice, respectively. There were no significant differences between OVA/OVA/vehicle mice and OVA/OVA/NEt-4IB mice. Additionally, there were no significant differences in liver size between the two groups (data not shown).

### Treatment with NEt-4IB suppresses NF-κB expression in the lung tissues

**Fig. 4:**
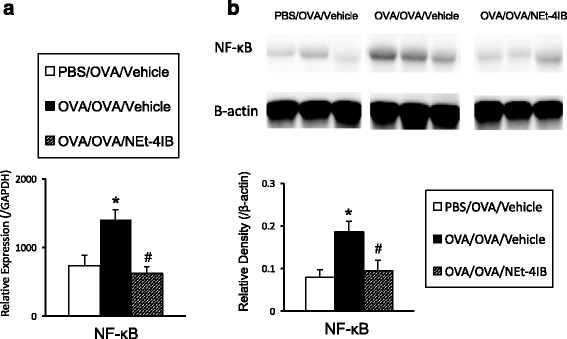
Treatment with NEt-4IB suppresses NF-κB expression in the lung. The expression levels of NF-κB were determined by RT-PCR (**a**) and Western blotting (**b**) as described in the Methods. *n* = 4 in each group * Significant differences (*P* < 0.05) between PBS/OVA/Vehicle and OVA/OVA/Vehicle. # Significant differences (*P* < 0.05) between OVA/OVA/Vehicle and OVA/OVA/NEt-4IB

NF-κB is a transcription factor that acts as a key regulator of immune and inflammatory genes, and activation of the NF-κB pathway has been implicated in asthma in both experimental models and humans [34, 35]. The NF-κB mRNA and protein levels were significantly increased in OVA/OVA vehicle-treated mice compared to PBS/OVA mice. Treatment with this partial agonist significantly attenuated the levels of NF-κB compared with OVA/OVA vehicle-treated mice (Fig. 4).

## Discussion

RXRs function not only as homodimers but also as permissive heterodimers with other NR superfamily members, including VDR, PPAR, and LXR. Therefore, an RXR agonist may exert potential effects on both RXRs and PPAR/RXR or LXR/RXR. A PPARγ agonist ameliorates experimental autoimmune encephalomyelitis or human central nerve system demyelinating diseases in animal models [36]. Additionally, agonists of PPARγ, LXR, and NR4A have some effects on allergic inflammation [16–18]. However, the role of RXR in mouse models of asthma remains unclear.

RXR full agonists are reported to be effective in the treatment of several diseases. In particular, in humans, Bexarotene is used for the treatment of cutaneous T-cell lymphoma [8, 37]. However, RXR full agonists are associated with strong adverse events, including the elevation of blood triglycerides, hepatomegaly and hypothyroidism [12, 13]. Accordingly, we developed a novel RXR partial agonist that displays no significant adverse events even with 28 consecutive days’ administration compared with full agonists [14]. In this study, elevations of TG levels and liver size were not recognized in OVA/OVA/NEt-4IB mice compared with OVA/OVA/vehicle mice. These data suggest that treatment with NEt-4IB in allergic inflammation is effective without severe adverse events.

In the present study, we demonstrated for the first time the potential of our novel RXR selective partial agonist NEt-4IB to present AHR and airway inflammation. Our results clearly show that AHR was significantly suppressed in NEt-4IB-treated mice in a dose-dependent manner, with attenuation of not only eosinophilic airway inflammation and Th2 cytokine production in the BAL fluid and goblet cell metaplasia in the lung but also TNF-α levels in the lung. These data also suggest that RXR plays critical roles in allergen-induced allergic inflammation.

We previously demonstrated that both CD4^+^ T cells and CD8^+^ T cells play pivotal roles in the development of AHR and airway inflammation [26]. In our study, treatment with NEt-4IB significantly decreased the number of CD8^+^ T cells as well as CD4^+^ T cells compared with vehicle-treated mice. Furthermore, the numbers of CD11b^+^ cells which are recognized as monocytes/macrophages and to a lower extent on granulocytes, NK cells, and a subset of dendritic cells in the lung were significantly lower in NEt-4IB treated mice.

To assess the mechanism of NEt-4IB, we performed in vitro experiments. The levels of IL-5 and IL-13 in the culture supernatant in OVA-re-stimulated lung MNCs isolated from OVA-sensitized and -challenged mice were significantly suppressed in OVA/OVA/NEt-4IB mice compared with OVA/OVA/vehicle mice. IL-5 and IL-13 levels in the OVA-re-stimulated spleen MNCs isolated from OVA-sensitized mice were significantly increased, and treatment with NEt-4IB in vitro also significantly attenuated the production of IL-5 and IL-13 in the culture supernatants in a dose-dependent manner. Moreover, there was no significant difference in Th2 cytokine production between the NEt-4IB (10 μM)-treatment group and PSL (1 μM)-treatment group. These results suggest that treatment with NEt-4IB suppresses Th2 cytokines both in the sensitization phase and effector phase and may be effective in regulating Th2-related allergic inflammation.

TNF-α is a pro-inflammatory Th1 cytokine that induces airway inflammation and hyperresponsiveness, mucus hypersecretion, and the activation of macrophages. In two small, placebo-controlled, double-blind, crossover studies of patients with corticosteroid refractory asthma, treatment with anti-TNF-α antibody (etanercept) resulted in clinical improvement in PC20, quality-of-life scores, and lung function [38] and improvement in asthma control questionnaire scores with reductions in the use of nebulized β2 agonists [39]. However, in a subsequent larger study in uncontrolled severe asthma, the anti-TNF-α antibody had no overall beneficial effects and increased the risk of serious side effects, although a post-hoc analysis suggested that patients with substantial bronchodilator reversibility had fewer exacerbations [40, 41]. These data may indicate the importance of TNF-α in the innate immune responses and suggest that complete suppression of TNF-α may be a critical and problematic approach. However, NEt-4IB is a partial RXR agonist, and treatment with NEt-4IB significantly suppressed TNF-α levels in the lung compared with vehicle-treated OVA/OVA mice in this study.

TNF-α triggers the activation of the inhibitor kappa-B (IκB) kinase (IKK)/NF-κB and mitogen-activated protein kinase (MAPK)/AP-1 pathways [42], and NF-κB translocation from the cytoplasm to the nucleus and binding to the promoter modulate the expression of inflammatory genes, including iNOS, inflammatory cytokines and chemokines. Additionally, several studies have reported that the activation of the NF-κB pathway is involved in both experimental murine asthma models and human asthmatics [34, 35]. Furthermore, iNOS produces nitric oxide (NO), which acts as a toxic radical and can cause tissue and cell damage. The over-production of NO may recruit eosinophils into the airway, resulting in the aggravation of airway inflammation [43]. Na et al. reported that RXR may directly modulate NF-κB-DNA interactions by forming a complex with NF-κB that cannot bind to κB sites [44]. In the present study, treatment with NEt-4IB significantly reduced NO levels in BAL fluid as well as TNF-α in lung homogenates compared with OVA/OVA vehicle-treated mice. These data suggest that NEt-4IB may affect eosinophilic inflammation by decreasing NO via the TNF-α/NF-κB pathway or directly interacting with NF-κB.

## Conclusion

These findings indicate the therapeutic potential of the novel RXR partial agonist NEt-4IB in the development of allergic airway inflammation and AHR. Although additional studies will be required to demonstrate that NEt-4IB may partially and permissively effect heterodimeric partners such as PPARs, NEt-4IB may be a promising candidate for the treatment of allergic airway inflammation and airway hyperresponsiveness in Th2 high allergic asthma.

## Abbreviations

AHR: Airway hyperresponsiveness
BAL: Bronchoalveolar lavage
iNOS: Inducible nitric oxide synthase
MCh: Methacholine
NO: Nitric oxide
NR: Nuclear receptor
OVA: Ovalbumin
RXR: Retinoid X receptor
